# Temporal information extraction from mental health records to identify duration of untreated psychosis

**DOI:** 10.1186/s13326-020-00220-2

**Published:** 2020-03-10

**Authors:** Natalia Viani, Joyce Kam, Lucia Yin, André Bittar, Rina Dutta, Rashmi Patel, Robert Stewart, Sumithra Velupillai

**Affiliations:** 1 Institute of Psychiatry, Psychology and Neuroscience, King’s College London, De Crespigny Park, London, SE5 8AF UK; 2 South London and Maudsley NHS Foundation Trust, London, UK

**Keywords:** Natural language processing, Electronic health records, Temporal information extraction, Schizophrenia, Mental health

## Abstract

**Background:**

Duration of untreated psychosis (DUP) is an important clinical construct in the field of mental health, as longer DUP can be associated with worse intervention outcomes. DUP estimation requires knowledge about *when* psychosis symptoms first started (symptom onset), and when psychosis treatment was initiated. Electronic health records (EHRs) represent a useful resource for retrospective clinical studies on DUP, but the core information underlying this construct is most likely to lie in free text, meaning it is not readily available for clinical research. Natural Language Processing (NLP) is a means to addressing this problem by automatically extracting relevant information in a structured form. As a first step, it is important to identify appropriate documents, i.e., those that are likely to include the information of interest. Next, temporal information extraction methods are needed to identify time references for early psychosis symptoms. This NLP challenge requires solving three different tasks: time expression extraction, symptom extraction, and temporal “linking”. In this study, we focus on the first step, using two relevant EHR datasets.

**Results:**

We applied a rule-based NLP system for time expression extraction that we had previously adapted to a corpus of mental health EHRs from patients with a diagnosis of schizophrenia (first referrals). We extended this work by applying this NLP system to a larger set of documents and patients, to identify additional texts that would be relevant for our long-term goal, and developed a new corpus from a subset of these new texts (early intervention services). Furthermore, we added normalized value annotations (“2011–05”) to the annotated time expressions (“May 2011”) in both corpora. The finalized corpora were used for further NLP development and evaluation, with promising results (normalization accuracy 71–86%). To highlight the specificities of our annotation task, we also applied the final adapted NLP system to a different temporally annotated clinical corpus.

**Conclusions:**

Developing domain-specific methods is crucial to address complex NLP tasks such as symptom onset extraction and retrospective calculation of duration of a preclinical syndrome. To the best of our knowledge, this is the first clinical text resource annotated for temporal entities in the mental health domain.

## Background

In the field of mental health, investigating the duration of untreated symptoms in relation to intervention outcomes represents an important research topic [1]. For patients with a diagnosis of schizophrenia, for example, the duration of untreated psychosis (DUP) is a widely used construct in research cohorts, defined as the period of time between first symptom onset and initiation of adequate treatment. A longer DUP has been linked to poorer cognitive function at the time of first presentation [2], and subsequently predicts more severe symptoms and greater social and functional impairment [3]. Therefore, routine identification of DUP across large clinical groups is a crucial step for prognostic monitoring and could form the basis for nested interventions to improve both clinical and functional outcomes at a service or population level. Electronic health records (EHRs) represent a valuable resource for large-scale retrospective clinical studies, as they contain a large amount of routinely collected patient data. In mental health services, however, relevant information on DUP is documented mainly in text fields and cannot therefore be easily analyzed automatically. To make this information available for computational analysis and clinical research, Natural Language Processing (NLP) methods can be used [4, 5].

EHR databases are typically large and complex, containing data for all patients in a clinical catchment area. Each patient may have varying levels of contact with health services, forming different trajectories and sets of EHRs. As a first step to develop real-world NLP applications using EHR data, it is important to identify appropriate documents for NLP development, i.e., those that are likely to include the information of interest. In the case of DUP extraction, relevant information would typically be documented in EHRs for patients with a psychotic disorder diagnosis: particularly in initial clinical assessment notes or paragraphs describing the patient’s previous clinical history or early psychosis symptoms, recorded around the time of first presentation and assessment. Once a relevant set of EHR documents (corpus) is defined, NLP techniques can be used to identify mentions of relevant symptoms as well as the associated temporal details. This represents a temporal information extraction challenge, which typically requires three different steps: (i) the identification of time expressions (*May 1st*), (ii) the identification of relevant concepts, such as symptoms (*hallucinations*) and treatments (*antipsychotic*), and (iii) the identification of temporal relations between entity pairs (*hallucinations* BEFORE *antipsychotic*), also known as temporal “linking”.

In recent years, manually annotated corpora and methods for temporal information extraction have been developed mainly based on the TimeML specification language, which was originally created for the general NLP domain (e.g., newspaper text) [6]. In the clinical domain, few gold standard corpora have been created and made available for temporal NLP development. Moreover, they address temporal modelling on a general level, without being driven by a specific clinical use-case such as DUP. Within the Informatics for Integrating Biology and the Bedside (i2b2) project, 310 de-identified discharge summaries from an intensive care unit were manually annotated for events, time expressions, and temporal relations [7]. This corpus was used in the 2012 i2b2 Challenge on temporal relation extraction, which required participants to develop NLP solutions to automatically extract these temporal elements [8]. In the oncology domain, Styler and colleagues created an annotated corpus of 1254 de-identified EHR notes, including both clinical and temporal information (the THYME corpus) [9]. This corpus consists of two types of EHR notes: clinical notes, which often include clearly delineated sections describing past and present events, and pathology reports, which contain a detailed analysis of specimens (taken at a single moment in time). Subsets of the THYME corpus were reused in different NLP challenges, among which Clinical TempEval 2015 and 2016 focused on temporal information extraction (440 and 591 documents, respectively) [10, 11]. In both i2b2 2012 and THYME, four main TimeML types of time expressions are defined: Date (e.g. *2011, yesterday*), Duration (e.g. *3 years, 1 week*), Frequency/Set (e.g. *daily*, *twice a week*), and Time (e.g. *10 am, the morning*). The THYME corpus also includes two additional types specific to the oncology domain: PrePostExp (expressions indicating Pre- and Post-operational concepts, e.g. *postoperative day #4*) and Quantifier (e.g. *twice, four times*).

Time expression extraction involves not only identifying textual spans representing time references, but also assigning a standardized value to them (*normalization*), which is crucial for anchoring clinical concepts on a patient timeline. In general, normalizing time expressions is a challenging task, especially due to the usage of relative (e.g. *2 days before*) and underspecified (e.g. *at 9 pm*) time expressions [12], as well as imprecise time expressions (e.g. *several weeks*) [13]. In the i2b2 2012 challenge, for example, 37% of all combined Date and Time expressions were relative: when evaluating the top 10 performing systems on these, the normalization accuracy dropped from around 0.67 to 0.32 [12]. Various strategies have been employed to tackle this difficulty. The SUTime system normalizes all relative expressions by comparison to the document creation time (DCT) [14]. The HeidelTime system uses rules and heuristics based on the document domain (e.g., news, narrative) to select anchor time expressions [15]. When evaluated on the i2b2 2012 dataset, teams using SUTime and HeidelTime achieved a normalized value accuracy of 0.54 and 0.6, respectively on the test set [8]. For this particular task, the highest normalization accuracies were obtained by using regular expressions (0.73) [16] and combining rules with supervised classifiers (0.72) [17]. To specifically deal with relative and incomplete expressions, Sun et al. combined multi-label classifiers for anchor points and anchor relations, achieving an improvement on relative and incomplete time expression value normalization from 0.45 (the top score on these expressions) to 0.54 [12]. As another interesting approach, the TimeNorm system relies on a synchronous context free grammar, which showed promising normalization performances on general domain datasets: on the TempEval 2013 dataset [18], for example, the reported normalization accuracy is 0.82 for TimeNorm, and 0.79 for HeidelTime [19].

**Fig. 1:**
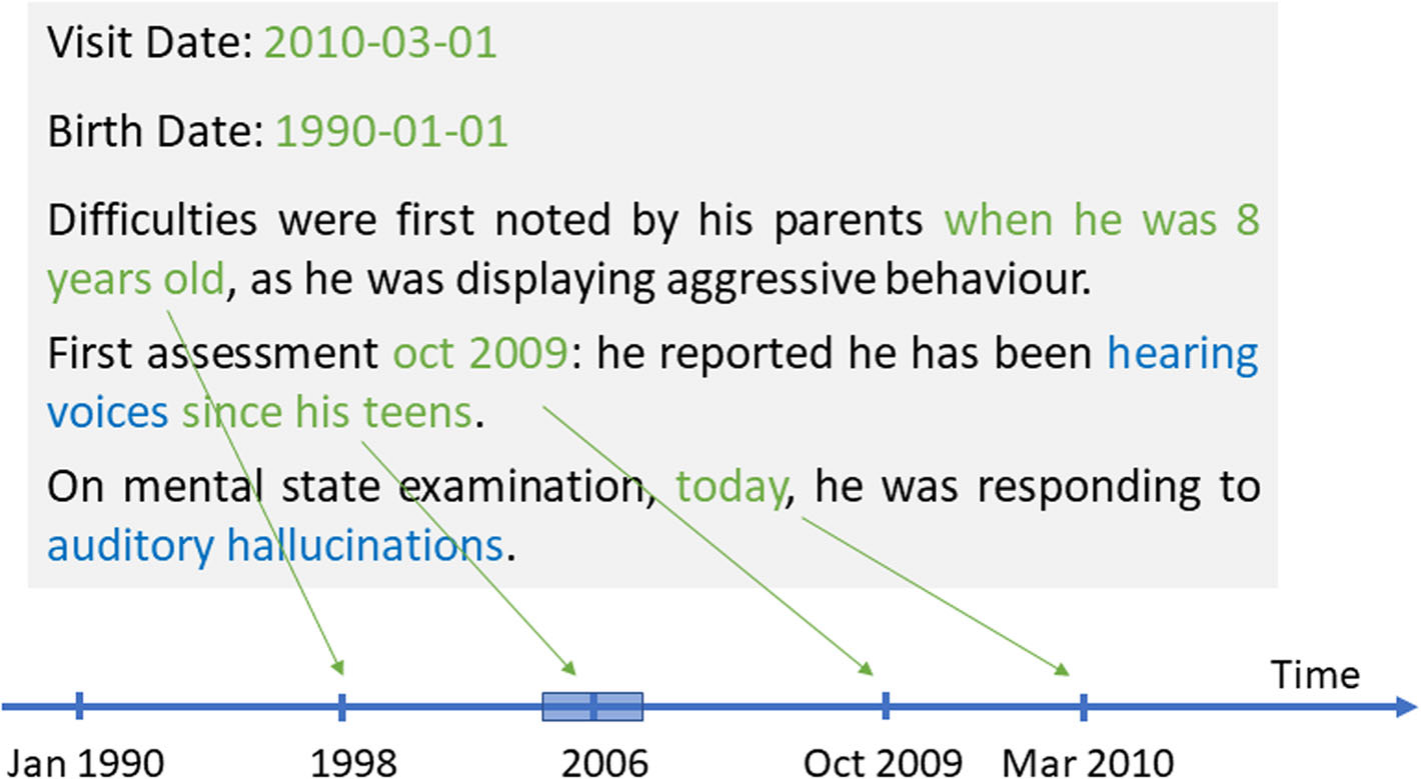
Example of clinical text describing the onset of psychosis symptoms. The example includes two structured dates (visit date and birth date) and four time expressions that are written in the text (“when he was 8 years old”, “oct 2009”, “since his teens”, “today”). As shown in the figure, time expressions can be normalized and placed on a timeline in order to reconstruct patient trajectories

Compared to other clinical domains, mental health records are characterized by a greater amount and variety of narrative portions, describing clinical histories and health assessments without necessarily relying on pre-defined structured sections containing a temporal anchor point. In this framework, relevant temporal information on DUP (e.g., associated to symptom onset or treatment initiation) is not always well represented by temporal models relying on TimeML. In previous work, to further investigate this aspect, we annotated a corpus of mental health documents for time expression spans and types, with a specific focus on patients with a diagnosis of schizophrenia [20]. Comparing this annotated corpus to two related works (the i2b2 2012 challenge and Clinical TempEval 2016), we found that mental health documents are much longer, with an average of 3974 tokens per document (vs. 574 for i2b2 2012 and 931 for Clinical TempEval 2016), and contain a larger variety of temporal references (including information taken from structured forms that is not relevant to the patient’s clinical history). Moreover, while notes in other domains can contain semi-structured date information (e.g. admission, discharge, or section dates), mental health texts often include various paragraphs describing both past and current events related to the patient. As regards information on DUP, from that work, we concluded that age-related expressions like *at age 8* or *in his teens* are important to temporally anchor the first occurrence of psychosis symptoms (Fig. 1). To capture these cases, we introduced a new type of time expression, Age_related, which represented 8.9% of all annotations in that corpus. We also evaluated, adapted and refined an existing rule-based system, SUTime [14], to extract time expressions in this domain.

Our long-term goal is to automatically extract from mental health notes all the elements needed for the generation of DUP data on a large patient cohort. To address this long-term goal, we have previously developed a corpus annotated with time expressions and adapted a time expression extraction system (SUTime) [20] to be used for temporal NLP development in the mental health domain - in particular to support DUP extraction [21]. Here, we extend this work with the following main contributions:

1. *Large-scale use of an adapted time expression extraction system for automated identification of relevant EHR documents:* We applied the adapted SUTime system on a large set of EHR documents from early intervention services for psychosis, to identify additional documents that would be relevant for calculating DUP.
2. *Time normalization annotation:* We extended our previous annotation effort on first referral EHR documents by adding the normalized values of the time expressions we had annotated in that corpus [20]. Furthermore, we annotated a new subset of documents from early intervention services, manually identifying time expression spans and values (without marking types).
3. *Automatic normalization*: We used the finalized annotated corpus to further refine our NLP time expression extraction system, with a focus on the normalization task. We also assessed the performance of this system on the i2b2 2012 dataset, to highlight key differences between the annotation tasks.

## Methods

### Dataset

In this study, we used data from the Clinical Record Interactive Search (CRIS) resource [22], which is derived from the EHR system adopted by the South London and Maudsley National Health Service (NHS) Foundation Trust (SLaM). Within CRIS, de-identified patient EHRs are rendered available for research within a robust governance framework. These include information from both structured fields and free text fields in the source EHR, the latter including case notes and clinical correspondence with automatically blanked-out identifiers [23]. In general, textual documents do not follow a specific structure and contain different types of patient information, e.g., past history, family history, examination results, and drug prescriptions. Moreover, although a document date can be retrieved from CRIS, it does not necessarily correspond to the actual document creation time (DCT), as there might be a temporal gap between the document creation and its upload to the system.

Relevant documents for calculating DUP would include a description of the patient’s clinical history or an assessment of early psychosis symptoms. Following advice from domain experts, two CRIS-derived datasets were considered:

- *First referral documents for schizophrenia patients*. In previous work, we extracted 52 early documents^1^ for patients with a diagnosis of schizophrenia [20]. For each patient, we considered the longest document, on the assumption that this first, long referral document would include the richest description of the patient’s clinical history. We call this dataset the *First referral corpus*.
- *Documents from early intervention services for psychosis*. We extended the set of documents to be used for annotation and NLP development, considering attachments from early intervention services for psychosis, i.e., mental health services that support people who are experiencing untreated psychosis for the first time. We call this dataset the *Early intervention corpus*.

As a first step to extend our *First referral* corpus, we extracted all clinical correspondence attachments from early intervention services for psychosis. For each patient, we considered the documents written within a 3-month window from the team’s acceptance date (36.6 k documents for 4166 patients). Because some of these documents were relatively short or contained forms or questionnaires rather than descriptive assessments, we filtered the initial set by only keeping longer documents, which were more likely to include information on symptom onset. More specifically, we calculated the document and line length on the entire document collection, and kept documents with length (in terms of characters) greater than the 50th percentile (2000 characters) and average line length greater than the 25th percentile (30 characters).

### Large-scale use of an adapted time expression extraction system for relevant EHR document identification

Through manual review of a small subset from the *Early intervention corpus,*^2^ we identified two criteria that could be useful in filtering documents. Firstly, we observed that documents containing relevant symptom keywords (e.g. *hallucinations*, *delusions*) were more likely to include passages on a patient’s clinical history and thus potentially symptom onset information. For this analysis, we used a list of 26 keywords that was defined by two psychiatrists^3^ (the list is publicly available [24]). Secondly, by applying our adapted version of SUTime on the same texts, we found that the number of extracted time expressions was in general higher in the documents mentioning symptom onset. Therefore, we applied this system on all available early intervention documents, using these criteria as additional filtering steps in order to maximize the amount of relevant documents for calculating DUP and minimize the number of irrelevant documents for costly manual review. More specifically, we kept all the documents containing at least one psychosis symptom keyword, and more than 5 time expressions, as we estimated this threshold would allow us to retain only the most relevant documents (based on our manual review).

### Manual time expression normalization

The *First referral* corpus consisted of documents that we had previously annotated for five types of time expressions (without normalized values): Date, Duration, Time, Frequency, and Age_related [20]. One of the findings in that study, through inter-annotator agreement (IAA) analysis on type classification, was that distinguishing between Date and Duration caused the most annotation disagreements (42%). For example, the expression “*last week*” was interpreted as a point in time (Date) by one annotator, and as a period of time (Duration) by another.

In the *First referral* corpus, we extended the time expression annotations by adding normalized values (“value” attribute), mostly following the TimeML specification language. To simplify the assignment of normalized values on difficult instances of Date/Duration time expressions, we instructed the annotators to keep prepositions within the marked text spans. In summary, the normalization rules were the following:

- Dates were normalized as “YYYY-MM-DD”, “YYYY-MM”, or “YYYY”, depending on their granularity. We also considered vague expressions such as *in the past* (value = PAST_REF) and *now* (value = PRESENT_REF).
- Times were normalized as “YYYY-MM-DDThh:mm”, when the date was available, or “XXXX-XX-XXThh:mm”, otherwise. We also considered times related to a time of the day, e.g. *in the morning* (TMO) and *at night* (TNI*)*.
- Durations and frequencies were normalized in the form “P (T)? Digit Granularity”, e.g., *for 4 years* (value = P4Y), *for 15 min* (value = PT15M).
- For durations marking a range of time, we added this information in the normalized value, considering two main cases. Expressions preceded by temporal prepositions like “since” and “until” (e.g. *since 2007, until a year ago*), which were common in our dataset, were normalized using the strings SIN and UNT (e.g. SIN2007, UNT2018). For explicit ranges denoted by a start point and an end point, e.g. “*2009–2012*”, we used the Duration type (rather than two separate Dates) with a brackets notation, e.g. value = (2009, 2012). This choice was made to keep the information on both the actual duration and the two endpoints.
- Similar to TimeML, vague durations (e.g. *for hours*) were normalized with a placeholder X (value = PXH).
- Age_related expressions were normalized in a similar way to standard durations, distinguishing between two different cases: expressions indicating the current age of the patient (e.g. a *45 year old* man, value = **P**45Y), and those referring to a previous point in time (e.g. *when he was 15*, value = **A**15Y). In addition, vague references like *when he was a child* were normalized with specific categorical values: CHILD_REF, SCHOOL_REF, TEENS_REF, ADULT_REF, UNI_REF, and OTHER_REF (for other cases).

To assess the impact of type classification on IAA values, the *Early intervention* corpus was only annotated with time expression spans and normalized values, without requiring specifying a type.

### Automated time expression extraction system refinement

Once the two corpora were annotated, we used the time expressions that were annotated with the same value by both annotators to further adapt and refine our automated time expression extraction system. We added post-processing rules on top of the default SUTime normalized values, with a focus on Age_related expressions and durations including prepositions. To develop these rules, we manually reviewed annotations from the *First referral* corpus development set used in our previous work (10 first referral documents, Table 1).

**Table 1:** Manual annotation results on the two EHR corpora (*First referral* and *Early intervention*)

Corpus	Batch	Documents (# tokens)	All annotations (A1, A2)	Overlapping annotations	Same value	IAA (acc)
First referral	dev	10 (49 K)	932, 972	913	768	0.84
First referral	valid	23 (83 K)	1455, 1475	1429	1254	0.88
First referral	test	19 (74 K)	1119, 1159	1100	927	0.84
Early intervention	batchA	14 (18 K)	435, 391	353	300	0.85
Early intervention	batchB	35 (57 K)	867, 822	714	600	0.84

Manual annotation results on the two EHR corpora (*First referral* and *Early intervention*) divided into development (dev), validation (valid) and test sets, and batches (batchA and batchB), respectively. IAA: Inter-annotator agreement; A1/A2: annotators 1 and 2

### Evaluation

To measure the IAA for time expression extraction, we computed the lenient F1 score, where a true positive (*match*) is defined as a textual span identified by both annotators (allowing overlapping spans). For the normalization task, we calculated accuracy (*acc*) only on matching spans, counting the proportion of expressions normalized with the same value.^4^ The system’s normalization performance was evaluated on these expressions, using the same metric (value accuracy for system true positives).

To highlight the specificities of our annotation task, we also applied our adapted time expression extraction system to the i2b2 2012 test set, manually analyzing errors and key differences in the two corpora.

## Results

### Large-scale use of adapted time expression extraction system

**Fig. 2:**
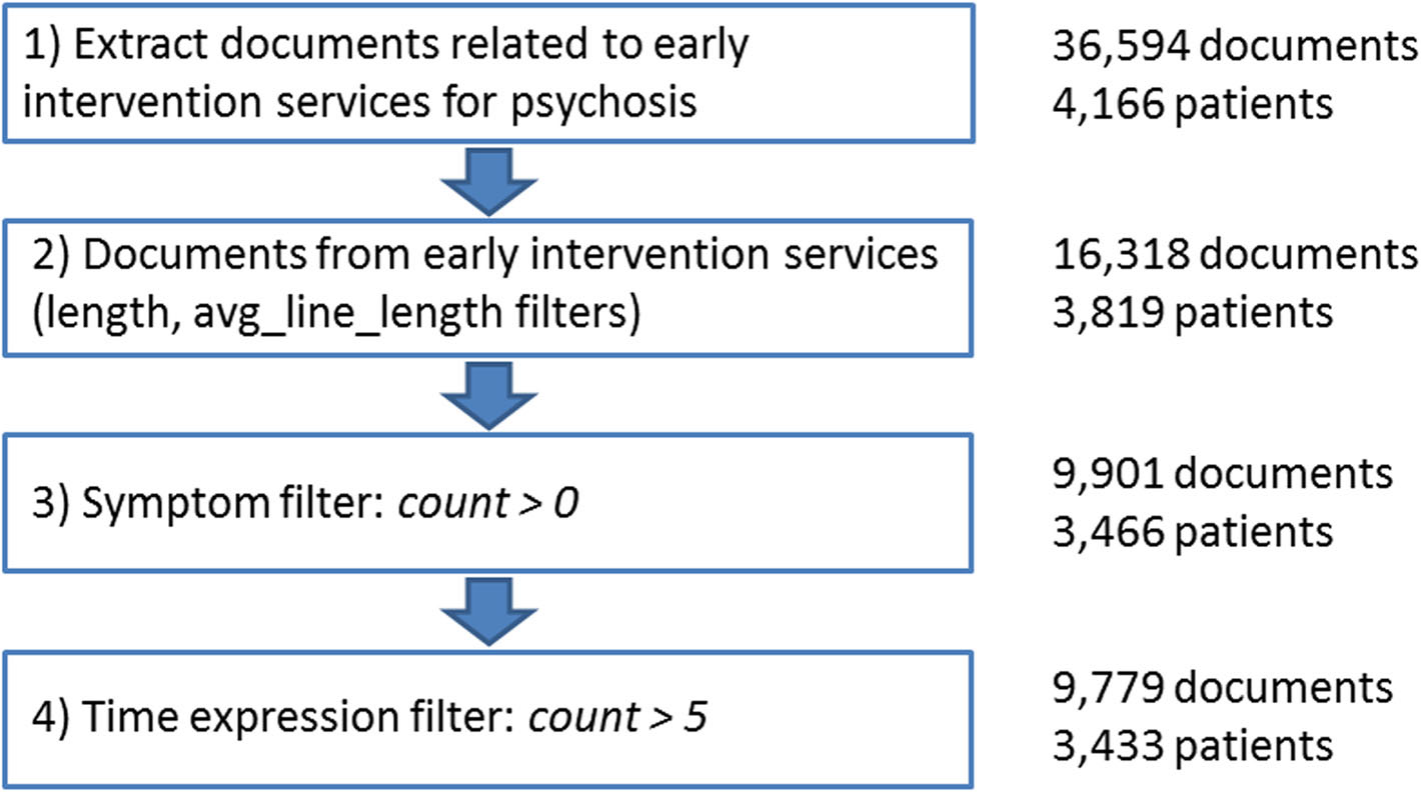
Filtering steps from EHR documents related to early psychosis intervention services. First, we retain documents with length and average line length (avg_line_length) greater than a certain threshold. Then, we keep documents including at least one psychosis symptom keyword (from a list of predefined keywords). Finally, we retain documents containing more than five time expressions (as automatically extracted by a rule-based system)

To obtain relevant documents for DUP extraction, the early intervention services dataset was filtered at different levels (Fig. 2) [21]. Note that the order of applying these filtering steps is not important.

**Fig. 3:**
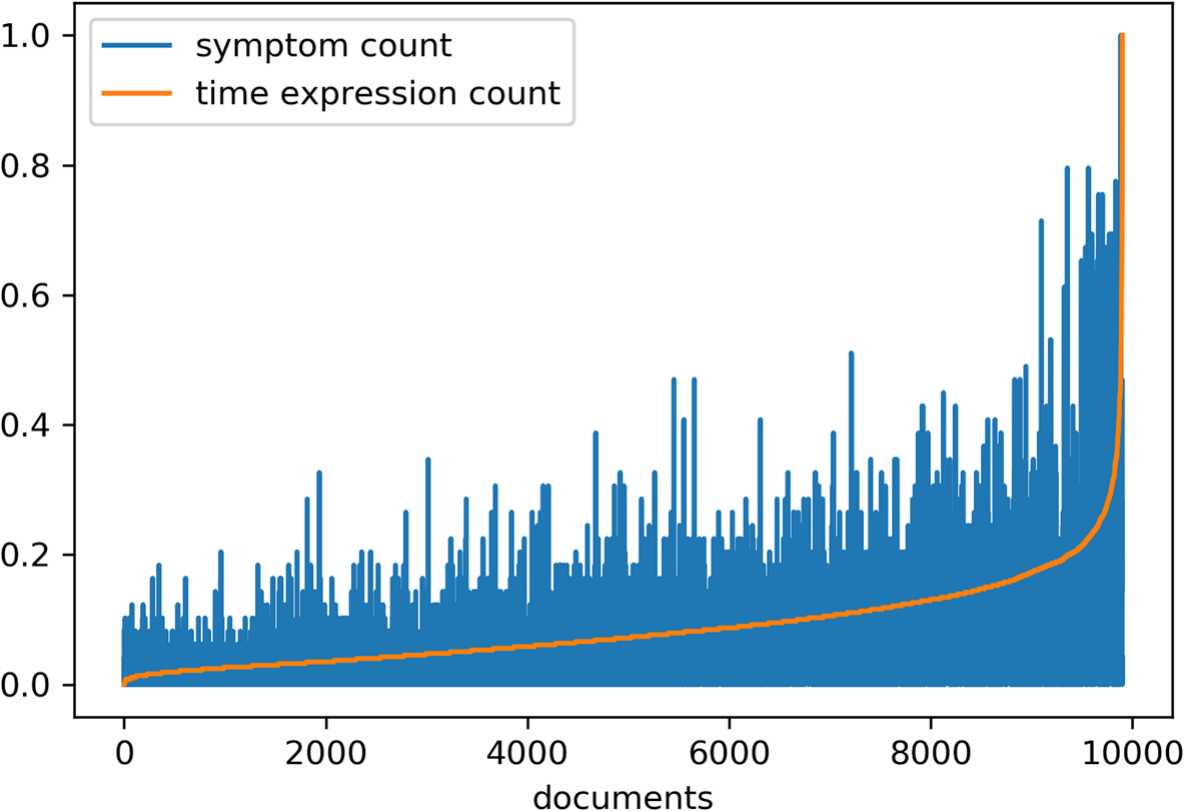
Psychosis symptom keyword and time expression counts in the early intervention services dataset. The x-axis represents the number of documents obtained after applying length, average line length, and psychosis symptom keyword filters (9901). The y-axis represents normalized counts for psychosis symptom keywords (blue) and automatically extracted time expressions (orange), normalized to the range 0–1. Texts containing many temporal expressions are more likely to also include relevant psychosis symptom keywords

We first removed short documents (length < 2000 characters) and texts consisting of short lines (average line length < 30 characters), which resulted in 16,318 documents for 3819 patients (i.e. around 55% of the initial documents were excluded, while 92% of the patients were retained). From these, we only retained the documents containing at least one relevant psychosis symptom keyword, which resulted in 9901 documents for 3466 patients (i.e. around 40% of the documents were filtered out, while 91% of the patients remained represented). Figure 3 shows the number of symptom mentions and time expressions found in these documents (normalized counts^5^): texts containing many temporal expressions are more likely to also include relevant psychosis symptom keywords. By also applying the SUTime-based filtering step, we obtained 9779 documents for 3433 patients (i.e. only about 2% of documents were further filtered out, retaining 99% of patients). We then randomly selected 20 of these patients for time expression and normalization annotation (49 documents).

### Manual time expression normalization

For the manual annotation task, the *First referral* corpus was pre-annotated with time expressions: in our previous study, the IAA on textual spans was 77% (lenient F1 score). The *Early intervention* corpus, on the contrary, was not pre-annotated. The resulting IAA on textual spans was 85%.

Table 1 shows the results of manual annotation for normalized values on both corpora. For the *First referral* corpus, we use the same data split as in previous work: development (*dev*), validation (*valid*), test (*test*). For the *Early intervention* corpus, we present results on two batches of 10 patients each (*batchA*, *batchB*). For both corpora, we report the number of documents, annotated time expressions (per annotator), overlapping time expressions, time expressions with the same normalized value, and the normalized value accuracy (the IAA measure).

In the development set, the most frequent type of disagreement was in the assignment of normalized values for relative expressions, such as *2 yrs back* (61/145), where it was hard to identify the anchor time in the text. Other disagreements involved the confusion between DCT and “PRESENT_REF” (e.g. *at this time*), non-standard dates (e.g., *week 3, over the weekend*), and time-of-the-day expressions (e.g. *at night*).

### Automated time expression value normalization system refinement

In the proposed automated time expression extraction system, time expression spans are first extracted with the adapted version of SUTime. Post-processing rules are then applied in order to improve the normalization step. Table 2 shows the performance of the developed system in normalizing values. The “reference standard” column represents the reference annotations, i.e., matched expressions where both annotators marked the same normalized value. The “TPs” column represents the time expressions that were correctly found by SUTime, which are used to compute the accuracy of the normalized values in the final system. First, we applied the adapted system as developed in previous work (“System1” column). Then, we created post-processing rules in order to improve the normalization step (“System2” column).

**Table 2:** Automated time expression normalization results on the two EHR corpora (*First referral* and *Early intervention*)

Corpus	Batch	Reference standard	TPs	System1 (value acc)	System2 (value acc)
First referral	dev	768	686	0.77	0.86
First referral	valid	1254	1115	0.76	0.80
First referral	test	927	828	0.66	0.71
Early intervention	batchA	300	272	0.76	0.86
Early intervention	batchB	600	556	0.82	0.86

Automated time expression extraction results (normalized values) on the two EHR corpora (*First referral* and *Early intervention*), divided into development (dev), validation (valid) and test sets, and batches (batchA and batchB), respectively. Accuracy values are reported on overlapping annotations (TPs) for both the first system (System1) and its refined version (System2)

The post-processing rules were developed to handle the following normalization values: 1) Age_related expressions, and 2) Duration expressions marking a range of time. In both cases, we defined specific keywords to be searched for in the corresponding string, e.g., “childhood”, “adolescence”. Moreover, we relied on keywords and regular expressions to disambiguate particular cases (e.g., current age of the patient vs. expressions referring to the past).

As shown in Table 2, the refinements performed in System2 were useful to improve normalization results, especially for the development set in the *First referral* corpus (accuracy from 0.77 to 0.86) and batches A and B in the *Early intervention* corpus (accuracy from 0.76–0.82 to 0.86). However, the improvement measured in the validation and test sets was lower (accuracy from 0.76 to 0.80, and from 0.66 to 0.71, respectively). To further investigate this, we analyzed IAA values and system accuracy per time expression type. The results of this analysis are shown in Table 3. Column “System2 (acc)” indicates that the major drop in performance was due to incorrect normalization of Time type expressions, for both the validation and the test sets. To determine the number of Time errors related to an underspecified Date part, we recalculated accuracy (acc*) by considering only the “Thh:mm” portion of the values normalized as “YYYY-MM-DDThh:mm”. For example, for the string *6 pm sharp*, the different values “2011–03-14 T18:00” and “2011–03-13 T18:00” would be considered as a match (value = T18:00). As shown in Column “System2 (acc*)”, this led to much higher accuracies also for the Time type (results marked in bold).

**Table 3:** Automated time expression normalization results on the *First referral* corpus, divided per time expression type

Batch	Type	IAA (matches)	IAA (acc)	System2 (TPs)	System2 (acc)	System2 (acc*)
dev	Date	572	0.84	427	0.93	0.93
	Time	77	0.87	65	**0.51**	**0.88**
	Duration	137	0.82	102	0.74	0.74
	Frequency	58	0.95	52	0.92	0.92
	Age_related	69	0.81	40	0.93	0.93
valid	Date	845	0.91	705	0.85	0.85
	Time	128	0.79	100	**0.27**	**0.64**
	Duration	209	0.77	147	0.84	0.84
	Frequency	123	0.98	101	0.95	0.95
	Age_related	124	0.81	62	0.73	0.73
test	Date	554	0.92	482	0.82	0.82
	Time	156	0.78	116	**0.09**	**0.78**
	Duration	192	0.72	128	0.77	0.77
	Frequency	90	0.72	48	0.83	0.83
	Age_related	108	0.86	54	0.80	0.80

Automated time expression extraction results (normalized values) on the *First referral* corpus (dev, valid, test), divided per time expression type. Results are presented in terms of inter-annotator agreement (IAA), system raw accuracy (System2 acc) and system relaxed accuracy (System2 acc*), where expressions with type Time are evaluated only on the “Thh:mm” portion

To gain more insight into system normalization performance, we also conducted a manual analysis of the 239 normalization value errors found in the *First referral* test set (from the 828 TPs, see Table 2). The majority of errors (110 expressions, 46%) originated from one particular document. This document had an unusual format with a EHR system-enforced structure with several mini-sections from some type of automated form with regular updates from an inpatient stay which included different paragraph dates – the system did not correctly use these as anchor dates, thus resulting in erroneous references for relative times (80/110) and dates (30/110). The second most frequent type of error was due to an incorrect structured DCT data field from the CRIS system (31 expressions, 13%) – in this case, normalized values were correctly extracted, but the provided structured DCT did not match what was written in the document. Other common errors were caused by relative expressions referred to previously mentioned dates (25 expressions, 10%), full dates not correctly recognized, e.g. *Friday 5 October 2012* (13 expressions, 5%), and periods not recognized, e.g. *since age 3 months* (13 expressions, 5%). Other important areas of improvement involve interval/ranges, e.g. *from 10 pm to 4 am* (9 expressions) and imprecise references, e.g. *for many years* (6 expressions). The remaining errors (32 expressions) did not form consistent patterns.

### Application of time expression extraction system to i2b2 2012 corpus

To further investigate the specificity of our corpus and annotation guidelines as compared to previous work in temporal NLP, we also applied our adapted time expression extraction system to the i2b2 2012 corpus, to analyze differences in time expression annotations and extraction performance. By applying the system on the 2012 i2b2 test set (120 documents), we obtained 0.71 F1-score and 0.47 value normalization accuracy, which is lower than the best performing systems in the 2012 i2b2 challenge: the top results for time expression extraction/normalization were 0.9/0.73, using regular expression pattern match and systematic reasoning [16], and 0.91/0.72, combining a CRF model and a context-free grammar algorithm [17]. When looking at the false negatives, two major differences were observed. First, the way dates are usually written in U.K. and U.S. clinical institutions is usually different (YYYY/MM/DD vs. YYYY/DD/MM), which impacts the system performance both in terms of extraction (full dates might not be recognized) and normalization. Second, the 2012 i2b2 corpus included annotations for time expressions related to clinical events, e.g. *at the time of discharge*, or *HD#2* (hospital day #2), which our corpus does not contain. Moreover, differently from the i2b2 2012 corpus, we annotated and implemented time expression extraction and normalization rules in our adapted SUTime for age-related expressions and imprecise references (e.g. present, past), which account for 15 and 32% of all false positives in the i2b2 2012 corpus.

## Discussion

In this study, we have made progress in addressing an ongoing challenge of automatically extracting DUP from mental health narratives. As a first step, we extended a previously annotated corpus of 52 first referral texts by including documents from early intervention services for psychosis. To keep only the documents that were relevant for our use-case, we used the output of a temporal information extraction system that we had adapted to the mental health domain. We then retained 49 of these documents (for 20 patients) for further annotation and NLP development. Both document subsets (first referrals and early intervention services) were annotated for time expression normalized values, mostly based on the TimeML specification language. As a final step, the manual annotations were used to refine our temporal information extraction system. The developed annotation guidelines are publicly available [24].

When applying NLP techniques to real-world clinical use-cases, selecting a suitable corpus for methods development is crucial. This is particularly true for complex problems such as DUP extraction, where the aim is to identify very specific information on a patient-level: this information could be documented only in a small portion of all patient-related texts, and it could be written in a variety of different ways. In our case, utilizing the developed temporal extraction system on a large dataset in combination with specific psychosis symptom keywords was useful to identify the documents containing information on the patient’s clinical history. This dataset is currently being analyzed for temporal relations between the identified time expressions and relevant symptom keywords, with the aim of capturing psychosis symptom onset information, which can then be used for DUP calculation.

Automatically extracting symptom onset for DUP calculation requires, among other NLP steps, the identification and normalization of temporal expressions. This is a challenging task in the mental health domain, especially due to the abundance of relative expressions, such as *3 years ago*, and imprecise age-related references, such as *when he was a child*. Referring relative expressions to the right anchor point is additionally difficult in EHR data, due to underlying procedures for document creation/upload – where the actual DCT is not always available. Moreover, the same document could contain different section/paragraph times that do not necessarily correspond to the stated document date. As for imprecise age-related references, we used categorical values for normalization (e.g. CHILD_REF or TEENS_REF). For subsequent DUP calculation, these would need to be associated to specific temporal ranges - relying on general world knowledge and shared definitions, e.g. (A13Y, A19Y) for TEENS_REF.

Despite the identified challenges, the availability of an automated system for capturing time expressions represents an essential step to anchor clinical concepts as accurately as possible. In the case of DUP extraction, the proposed system will be helpful to link the first onset of symptoms to the corresponding time period - even if the exact temporal reference is not exactly identifiable. Within the CRIS resource, the document/section dates - as written in the text - and the corresponding structured fields are not likely to differ by more than 1 month. On the contrary, early symptom onset is often documented in an imprecise way, which could lead to a more inaccurate estimation (with a year or more error). For this reason, we believe that differences in written vs. structured DCTs will not have a major impact on our approach for DUP calculation. Similarly, using a range notation to identify a patient’s period of life will be helpful to give an estimate of onset dates, where the actual value to be used for DUP calculation can be defined depending on the specific study. For example, it might be important to distinguish between current, recent and historical onsets (e.g., longer than 1 year) – keeping each mention explicitly related to a certain level of uncertainty.

In this study, we considered two subsets of documents from EHRs: first referral documents for patients with a diagnosis of schizophrenia and documents from early intervention services for psychosis. The first referral dataset was pre-annotated with adjudicated time expressions (including types), while the early intervention services dataset was not pre-annotated (in this case, only normalized values were required). The IAA on textual spans was higher in this second dataset compared to our previous work (85% vs. 77%), while the agreement on normalized values remained comparable (84–85% vs. 84–88%). This indicates that the annotation guidelines developed in previous work were useful even when applied to a different set of documents.

As regards the system error analysis performed on the i2b2 2012 corpus, we noticed some key differences between both the corpora and the time expression annotations. First, our adapted version of SUTime missed some full or underspecified dates, partly due to the different date formats that are used in U.S. and U.K. clinical notes. Moreover, we annotated and extracted age-related and imprecise temporal references, as these expressions were deemed as useful for contextual analysis of psychosis symptom mentions. Another important observation concerns the types of documents in the two corpora: the 2012 i2b2 corpus consists of discharge summaries only, while our corpus contains a variety of clinical document types.

Despite the inherent complexity of our normalization task, the preliminary time expression extraction and normalization system we developed provides reasonable performance. Future directions will concern the normalization of relative Time expressions, where anchor times are available inside the text, as well as further improvement on Duration extraction and normalization. The first issue could be addressed by changing the anchor date for each expression (in a similar way that the HeidelTime system deals with this), while the second issue could be partly improved by adding and refining rules. Moreover, we will investigate whether all mentioned dates/times are actually useful for clinical timeline reconstruction. For example, there are a number of documents (especially in *First referral* validation and test) which include “structured” section times that are not actually related to any clinically relevant information.

This study has some limitations. First, as regards the large-scale application of the adapted SUTime system, we did not verify whether the documents that were filtered out could still be useful for our long-term goal of extracting DUP information. Also, for documents that were excluded in the first step (length and average line length filters), we did not check the presence of symptom keywords and temporal expressions –we will investigate the impact of this in a future extension. Furthermore, for this particular use-case, in addition to accurately finding anchor points in time, appropriate psychosis symptom keywords are essential. Here, we have used a predefined set of terms developed by domain experts, which of course might be too restrictive. We are therefore also looking at methods to automatically extend these vocabularies using data-driven methods [25], and will investigate whether this could impact our proposed document filtering approach as well as downstream automated temporal reasoning steps. As observed, using the time expression count on top of the symptom keyword count did not identify many additional entries to be removed. To further assess the utility of the two filters (separately and in combination), we will further review a sample of these texts, to gain more knowledge on how both time expression and psychosis symptom keyword information is documented within the mental health EHR and how this relates to symptom onset information. As another limitation, the annotations in the *Early intervention* corpus were not manually adjudicated. For this reason, normalization results - which were evaluated on overlapping time expressions where the annotators marked the same value - might slightly change when evaluated on a larger set of adjudicated time expressions. Finally, in this study we only considered rule-based approaches for time expression extraction and normalization. In the future, we plan to investigate supervised machine learning methods and more data-driven approaches.

## Conclusions

Extracting DUP information from free text is an important step to improve large-scale research in mental health using the increasing volumes of EHR data currently accumulating. This NLP challenge requires different steps, for which developing domain-specific resources and methods is essential. In this study, we identified two relevant sets of EHR documents for our use-case, and annotated them for time expression spans and values - which are needed for an accurate representation of a patient’s timeline and, by extension, calculating DUP. We also adapted a rule-based system for time expression extraction and normalization in this domain. To the best of our knowledge, this is the first clinical data resource annotated for temporal entities in the mental health domain.

## Data Availability

The EHR-derived datasets generated and/or analyzed during the current study are not publicly available due to governance regulations. However, there are procedures in place to provide researchers with controlled access to CRIS, and thus the source data are available on request and subject to access approval.

## Abbreviations

DUP: Duration of untreated psychosis
EHR: Electronic health record
NLP: Natural language processing

## Footnotes

1 Documents were written within 3 months of patients’ first presentation to mental health services.

2 We manually reviewed a sample of 70 documents, marking relevant sentences such as “*his mother first expressed concerns about his behaviour when he was 8 years old*”.

3 Starting from a comprehensive list of psychosis symptoms, two psychiatrists reviewed the most frequent terms in the *Early intervention corpus*, thus agreeing on the final list of 26 keywords to be used as a filter.

4 For relative expressions like *1 year ago*, we did not consider “granularity” differences as errors (e.g., given a document creation time of 2012-01-01, *1 year ago* could be normalized as “2011” or “2011-01-01”).

5 All numbers were normalized to the range 0–1.

## References

[1] Kisely S, Scott A, Denney J, Simon G (2006). Duration of untreated symptoms in common mental disorders: association with outcomes. Br J Psychiatry.

[2] Lappin JM, Morgan KD, Morgan C, Dazzan P, Reichenberg A, Zanelli JW (2007). Duration of untreated psychosis and neuropsychological function in first episode psychosis. Schizophr Res.

[3] Hill M, Crumlish N, Clarke M, Whitty P, Owens E, Renwick L (2012). Prospective relationship of duration of untreated psychosis to psychopathology and functional outcome over 12 years. Schizophr Res.

[4] Meystre SM, Savova GK, Kipper-Schuler KC, Hurdle JF. Extracting information from textual documents in the electronic health record: a review of recent research. Yearb Med Inform. 2008;17(01):128–44. 18660887

[5] Wang Y, Wang L, Rastegar-Mojarad M, Moon S, Shen F, Afzal N (2018). Clinical information extraction applications: a literature review. J Biomed Inform.

[6] Pustejovsky J, Castano JM, Ingria R, Sauri R, Gaizauskas RJ, Setzer A (2003). TimeML: robust specification of event and temporal expressions in text. New Dir Quest Answering.

[7] Sun W, Rumshisky A, Uzuner O (2013). Annotating temporal information in clinical narratives. J Biomed Inform.

[8] Sun W, Rumshisky A, Uzuner O (2013). Evaluating temporal relations in clinical text: 2012 i2b2 challenge. J Am Med Inform Assoc.

[9] Styler WF, Bethard S, Finan S, Palmer M, Pradhan S, de Groen PC (2014). Temporal annotation in the clinical domain. Trans Assoc Comput Linguist.

[10] Bethard S, Derczynski L, Savova G, Pustejovsky J, Verhagen M (2015). SemEval-2015 Task 6: Clinical TempEval.

[11] Bethard S, Savova G, Chen W-T, Derczynski L, Pustejovsky J, Verhagen M (2016). Semeval-2016 task 12: Clinical TempEval.

[12] Sun W, Rumshisky A, Uzuner O (2015). Normalization of relative and incomplete temporal expressions in clinical narratives. J Am Med Inform Assoc.

[13] Tissot H, Del Fabro MD, Derczynski L, Roberts A. Normalisation of imprecise temporal expressions extracted from text. Knowl Inf Syst. 2019;61:1361–94.

[14] Chang AX, Manning CD. SUTIME: a library for recognizing and normalizing time expressions. In: Proceedings of the Eighth International Conference on Language Resources and Evaluation (LREC 2012); 2012. p. 3735–40.

[15] Strötgen J, Gertz M (2010). Heideltime: High quality rule-based extraction and normalization of temporal expressions. Proceedings of the 5th International Workshop on Semantic Evaluation.

[16] Sohn S, Wagholikar KB, Li D, Jonnalagadda SR, Tao C, Komandur Elayavilli R (2013). Comprehensive temporal information detection from clinical text: medical events, time, and TLINK identification. J Am Med Inform Assoc.

[17] Xu Y, Wang Y, Liu T, Tsujii J, Chang EI-C (2013). An end-to-end system to identify temporal relation in discharge summaries: 2012 i2b2 challenge. J Am Med Inform Assoc.

[18] UzZaman N, Llorens H, Derczynski L, Allen J, Verhagen M, Pustejovsky J (2013). SemEval-2013 Task 1: TempEval-3: Evaluating Time Expressions, Events, and Temporal Relations. Second Joint Conference on Lexical and Computational Semantics (*SEM), Volume 2: Proceedings of the Seventh International Workshop on Semantic Evaluation (SemEval 2013).

[19] Bethard S (2013). A synchronous context free grammar for time normalization. Proceedings of the 2013 Conference on Empirical Methods in Natural Language Processing.

[20] Viani N, Yin L, Kam J, Alawi A, Bittar A, Dutta R (2018). Time expressions in mental health records for symptom onset extraction. Proceedings of the Ninth International Workshop on health text mining and information analysis.

[21] Viani N, Kam J, Yin L, Verma S, Stewart R, Patel R (2019). Annotating temporal relations to determine the onset of psychosis symptoms. Stud Health Technol Inform.

[22] Perera G, Broadbent M, Callard F, Chang C-K, Downs J, Dutta R (2016). Cohort profile of the South London and Maudsley NHS Foundation Trust biomedical research Centre (SLaM BRC) case register: current status and recent enhancement of an electronic mental health record-derived data resource. BMJ Open.

[23] Fernandes AC, Cloete D, Broadbent MT, Hayes RD, Chang C-K, Jackson RG (2013). Development and evaluation of a de-identification procedure for a case register sourced from mental health electronic records. BMC Med Inform Decis Mak.

[24] Viani N, Velupillai S. Project repository. Available from: https://github.com/medesto/temporal-information-extraction-DUP.

[25] Viani N, Patel R, Stewart R, Velupillai S (2019). Generating positive Psychosis Symptom Keywords from Electronic Health Records. Proceedings of the 17th Conference on Artificial Intelligence in Medicine (AIME 2019).

